# Narrowing the knowledge gaps for melanoma

**DOI:** 10.3109/03009734.2012.658977

**Published:** 2012-04-19

**Authors:** Ana Slipicevic, Meenhard Herlyn

**Affiliations:** ^1^The Wistar Institute, Philadelphia, Pennsylvania, USA; ^2^Department of Pathology, The Norwegian Radium Hospital, Oslo University Hospital, Oslo, Norway

**Keywords:** Molecular biology, oncogenes, tumor biology

## Abstract

Cutaneous melanoma originates from pigment producing melanocytes or their precursors and is considered the deadliest form of skin cancer. For the last 40 years, few treatment options were available for patients with late-stage melanoma. However, remarkable advances in the therapy field were made recently, leading to the approval of two new drugs, the mutant BRAF inhibitor vemurafenib and the immunostimulant ipilimumab. Although these drugs prolong patients' lives, neither drug cures the disease completely, emphasizing the need for improvements of current therapies. Our knowledge about the complex genetic and biological mechanisms leading to melanoma development has increased, but there are still gaps in our understanding of the early events of melanocyte transformation and disease progression. In this review, we present a summary of the main contributing factors leading to melanocyte transformation and discuss recent novel findings and technologies that will help answer some of the key biological melanoma questions and lay the groundwork for novel therapies.

## Traditional targets and therapies

Melanoma is the most aggressive form of skin cancer, causing 50,000 deaths annually worldwide, and its incidence continues to increase. If the tumor is detected early, before it has invaded into the dermis, surgical excision provides a cure in about 99% of patients. However, the 5-year survival rate falls to 15% and to a median survival of 1 year for those with advanced, disseminated disease (1).

Until recently, there were few approved and successful treatment options for advanced-stage melanoma. Common treatment strategies included conventional chemotherapy with dacarbazine (DTIC) and the cytokines interleukin-2 (IL-2) and interferon-a2b (IFN-a2b). While only 5%–10% of the patients respond to DTIC treatment, IL-2 treatment with the use of IFN-a2b as adjuvant immunotherapy achieves a response in 10%–20% of patients (1). Unfortunately, these responses are generally short-lived and associated with high toxicity. Encouragingly, in recent years the identification of the main genetic aberrations and signaling pathways involved in melanocyte transformation and disease progression has resulted in the development of novel, more effective therapeutic approaches.

The first clues about the molecular basis of melanoma came from studies of families prone to the disease and the identification of two autosomal-dominant high-susceptibility loci: cyclin-dependent kinase inhibitor 2A (*CDKN2A*) and cyclin-dependent kinase 4 (*CDK4*). The *CDKN2A* locus encodes two tumor suppressor proteins, p16^INK4a^ and p14^ARF^. While p16^INK4a^ is an inhibitor of the cyclin-dependent kinases CDK4 and CDK6 and prevents S-phase entry during cell cycle, p14^ARF^ acts as a positive regulator of p53. Deletions of the *CDKN2A* locus have been found in up to 50% of melanomas, but inactivation of this locus can also occur through mutations and promoter hypermethylation (2-4). Although undoubtedly important for melanoma development, in terms of therapy, direct targeting and restoring of the function of tumor suppressor proteins have been inherently difficult.

Possibilities for novel therapeutic options came with the realization that the mitogen activated protein kinase (MAPK) pathway is a crucial regulator of melanoma development. In fact, activation of this pathway regulates both proliferation and survival of melanoma cells. The underlying mechanism of MAPK deregulation is attributed to activating mutations in *NRAS* and *BRAF* genes, resulting in a constitutive activation of the pathway. In addition, autocrine growth factors, adhesion molecule signaling, and morphogen signaling all contribute to MAPK pathway activation. While *NRAS* mutations are observed in 15%–25% of melanomas, *BRAF* is mutated in as many as 50% of the cases (5). In addition, more than 95% of the mutations in *BRAF* affect a valine residue at the 600 amino acid position (*BRAF^V600E^*), while the *NRAS* mutation is most often a glutamine-to-arginine substitution at position 61 (*NRAS^Q61R^*).

This increased knowledge about the molecular changes in melanoma led to the application of sorafenib, the first agent targeting the MAPK pathway. Disappointingly, in phase II trials, sorafenib demonstrated poor ability to inhibit MAPK pathway activation and little to no clinical efficacy as a single agent. The real advance came with the development of more potent and selective BRAF^V600E^ inhibitors including vemurafenib (PLX4032). Clinical results from phase I, II, and III trials showed that vemurafenib treatment caused complete or partial tumor regression in 80% of patients carrying BRAF^V600E^ tumors as well as an increased progression-free survival rate (6,7). Given this success, several other pharmacological inhibitors targeting proteins of the MAPK pathway, including MEK and ERK, are being developed (8). Unfortunately, after an initial period of response to vemurafenib, most patients relapse and develop resistance to the drug (7). Resistance can be acquired through several mechanisms including activation of the serine/threonine kinase COT and RAF isoform switching (9). Furthermore, we and others have shown that the elevation of insulin-like growth factor receptor (IGFR) or platelet-derived growth factor receptor (PDGFR) confer resistance to BRAF^V600E^ inhibitors through activation of the PI3K/Akt signaling pathway (10-12), collectively suggesting that targeting of the PI3K/Akt signaling pathway along with the MAPK pathway might provide a new and more effective therapeutic approach for melanoma treatment.

Melanoma represents a heterogeneous group of neoplasms with variable genotypic and phenotypic traits based on anatomic location, degree of sun exposure, and individual susceptibility. For instance, several genetic studies indicate that acral and mucosal melanomas develop through different etiological pathways than cutaneous melanomas. In these subtypes, the MAPK pathway is not activated through the same mutation as in the cutaneous counterparts. While *BRAF* mutations are significantly less frequent in these melanoma subtypes, activating mutations in the *KIT* gene are often seen. *KIT* encodes a receptor tyrosine kinase (c-Kit) that plays an important role in the development, proliferation, and survival of melanocytes (13). The therapeutic potential of targeting c-KIT in this subgroup of melanomas was validated by two clinical studies where patients with activating mutations in c-KIT showed significant responses to the c-KIT inhibitor imatinib (14,15); however, overall clinical responses to this inhibitor are less pronounced than to BRAF inhibitors.

Numerous other molecular alterations contribute to the complexity of melanoma biology, including mutations of receptor tyrosine kinases ERBB4 and EPH, activation of vascular endothelial growth factor receptors (VEGFR), deregulation of p53, MITF expression, and the developmental signaling pathways Notch and Wnt (16-19). However, attempts to target these pathways therapeutically have not been successful so far.

Improved understanding of tumor immunobiology has also provided novel treatment approaches for melanoma. Ipilimumab is a monoclonal antibody that augments T-cell activation and proliferation by blocking the cytotoxic T-lymphocyte antigen-4, a critical negative regulator of the antitumor T-cell response. In advanced-stage melanoma patients, treatment with ipilimumab resulted in a 20% increased survival up to 4 years after treatment (20). Nevertheless, only a fraction of patients receive durable benefits from ipilimumab therapy.

Thus, even though ipilimumab and vemurafenib have created enthusiasm in the melanoma therapeutic field, it is evident that further improvements are necessary. Likely, the key to further success in therapy lies in combination therapies, in which two or more drugs are combined. The lack of good therapeutic targets outside of the MAPK pathway also underlines the need for further analysis of currently known drivers of melanocyte transformation and melanoma progression as well as identification of new ones.

## The path to melanocyte transformation

Melanocytes develop during embryogenesis from melanoblastic precursors that migrate from the neural crest to populate the epidermis, hair follicles, cochlea, and the uveal tract of the eye (21). Once situated in the epidermis, melanocytes remain under tight control by keratinocytes and proliferate only after stimulation by paracrine factors secreted by the keratinocytes (22). Development and progression of melanoma depends on a set of genetic alterations that allow melanocytes to escape regulation by keratinocytes, migrate, invade, and survive in a ‘hostile’ environment. These alterations are termed ‘drivers’ of the disease. In addition to these alterations, the cells also acquire many secondary or ‘passenger’ alterations as a result of general genome instability, which is particularly seen in melanoma cells. The ability to distinguish the essential driver from passenger mutations is critical to the development of effective therapies.

**Figure 1.**
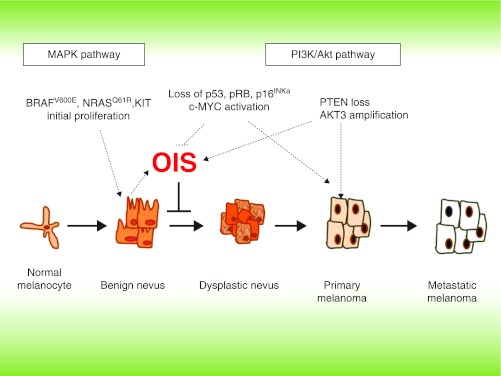
Genetic alterations leading to bypass of oncogene-induced senescence (OIS) and transformation of melanocytes.

Acquisition of the *BRAF^V600E^* mutation is considered an early event in the initiation of melanocytic neoplasia but in itself is not sufficient for full transformation (Figure 1). For example, overexpression of BRAF^V600E^ in melanocytes of transgenic zebrafish and mice induces nevus-like cell growth but does not lead to melanoma development (23,24). In normal human melanocytes (NHM), ectopic introduction of BRAF^V600E^ *in vitro* leads to cellular senescence, an irreversible form of cell cycle arrest (25). Senescence is often characterized by phenotypic features, such as increased senescence-associated β-galactosidase (SA-β Gal) activity and elevated levels of negative regulators of the cell cycle, such as p16^INK4a^, p53, and p21^CIP/WAF^ (25,26). Interestingly, both p16^INK4a^ and p53 were reported to be dispensable for BRAF^V600E^-induced senescence in NHM, and the exact mechanisms of this oncogene-induced senescence (OIS) are still not fully understood (27,28). However, it is assumed that OIS represents a major barrier to oncogenic transformation which makes OIS mechanisms important for further studies. Benign nevi, which are considered non-proliferative melanocyte lesions, represent the best *in vivo* evidence of OIS. *BRAF^V600E^* mutations are found in 60% of benign nevi and are thought to result in the initial increase in proliferation of melanocytic cells followed by induction of cell cycle arrest and senescence (29).

In addition to BRAF^V600E^, overexpression of constitutively active mutants of RAS, most commonly HRAS^G12V^ and NRAS^Q61R^, also leads to OIS in NHM. However, there are differences between mechanisms of OIS induction in each case. For example, HRAS^G12V^-specific senescence can be mediated by the endoplasmatic reticulum (ER)-associated unfolded protein response (UPR) (27). Moreover, p16^INK4a^, p14^ARF^, and p53 do not appear to play a prominent co-operating role in the HRAS^G12V^-induced senescence. In the case of NRAS^Q61R^, contribution of both p53 and pRb is evident due to the fact that depletion of either factor makes NHM refractory to senescence induction (30). Collectively, these observations underline mutation-specific differences in senescence induction. Interestingly, mutation specificity is also seen in melanoma cells and has to be taken into consideration when evaluating therapeutic approaches. In melanoma cells RAS mutations are not equivalent to BRAF mutations since they activate the MAPK pathway in different ways. While BRAF mutations result in direct phosphorylation and activation of MEK, RAS mutations induce a switch in signaling from BRAF to CRAF, allowing CRAF to signal to MEK (31,32).

Since approximately 30% of melanomas arise from nevi, these cells must have acquired additional alterations during the transformation process allowing them to overcome OIS. Recently it has been shown that the ectopic expression of the oncogenic transcription factor c-MYC can significantly suppress OIS in NHM (28). A similar effect is obtained by depletion of the B56α subunit of the PP2A tumor suppressor complex, which also leads to the up-regulation of endogenous c-MYC (33). Hömig-Hölzel et al. demonstrated that suppression of the transcription factor and putative tumor suppressor gene TGFβ-stimulated clone 22 (TSC22D1) allows NHM to bypass *BRAF^V600E^*-induced senescence (34). Another protein recently reported to modulate OIS in NHM is pirin, a highly conserved nuclear protein belonging to the cupin superfamily14. Down-regulation and overexpression experiments showed that pirin is involved in the negative control of OIS and that its expression is necessary to overcome the senescence barrier (35). In addition, pirin controls melanoma cell migration through the transcriptional regulation of snail homolog 2 (SNAI2) (36).

It is interesting to speculate whether acquisition of the alterations allowing melanocytes to bypass OIS is sufficient for the development of the malignant phenotype. In animal models, *BRAF^V600E^* induces melanoma when combined with p16^INK4a^/p14^ARF^ or p53 deficiency, as well as when combined with the activation of β-catenin that leads to silencing of p16^INK4a^ (24,37-40). However, Dhomen et al. recently reported that *BRAF^V600E^* can induce melanoma in mice without p16^INK4a^ inactivation. Still, these lesions appeared after a long latency period, and it is possible that additional unidentified genetic alterations have occurred during this time (23).

Previously it has been shown that NHM immortalized with the oncogenic simian virus 40 (SV40) large-T, human telomerase reverse transcriptase, and transduced with either HRAS^G12V^ or c-Met, gain the ability to form tumors *in vivo* (41). In addition, combined expression of MITF and BRAF^V600E^ also led to transformation of NHM (42). In our *in vitro* models, transformation of NHM without immortalization by the SV40 can be achieved by combining BRAF^V600E^ and inactivating both the p53 and Rb pathways (43).

Increasing evidence supports the hypothesis that activation of the PI3K/Akt pathway, which acts upstream of both β-catenin and c-MYC, may also be involved in melanocyte senescence suppression and transformation (28,44). Chudnovsky et al. showed that invasive human melanocytic neoplasia can be induced by activation of PI3K when combined with Rb and p53 inhibition as well as hTERT expression (45). Interestingly, in the same study, BRAF^V600E^ did not show the same oncogenic potential as PI3K, suggesting that the effect of different genes on transformation depends on specific combinations and the timing of mutational events as well as the experimental model used. In contrast, expression of BRAF^V600E^ combined with PTEN gene silencing induced melanomas with 100% penetrance, short latency, and metastatic disease in a mouse model (44). In NHM, however, PTEN deficiency combined with BRAF^V600E^ activation induced a melanoma *in situ*-like phenotype without dermal invasion in organotypic human skin culture. Only after further addition of cell autonomous TGFβ did these lesions develop an invasive phenotype (46).

In addition to the MAPK and PI3K/Akt pathways, other signaling networks are likely important for OIS escape and melanocyte transformation. The Notch signaling pathway, for example, plays a critical role in the proper development of melanocytes from neural crest precursors (reviewed in (47)). Upon ligand activation, Notch receptors are proteolytically cleaved, resulting in the liberation of the intracellular domain (NIC) that translocates into the nucleus and initiates transcription of target genes. Transduction of primary melanocytes with this intracellular domain leads to activation of the Notch signaling pathway and induces a transformed phenotype *in vitro* (48). However, NIC-transfected cells do not form tumors in NOD-SCID mice, suggesting that Notch overexpression in itself is not sufficient for neoplastic transformation.

## Future perspectives; new knowledge through new models

The identification of novel drivers of OIS escape and melanocyte transformation and the further characterization of melanoma based on genetic subgroups are critical for improving current therapies. In the last few years, complex melanoma genetics have been addressed by extensive genome-wide association studies (GWAS). By comparing frequencies of genetic polymorphisms, between melanoma patients and healthy individuals, these studies aim to identify novel melanoma susceptibility genes. Most of the studies have analyzed single nucleotide polymorphisms (SNPs).The main advantage of this approach is the capability for highly detailed and robust data collection. In melanoma, GWAS validated the importance of the well-known, high-penetrance alleles *CDKN2A* and *CDK4*, while identifying several novel low-risk alleles associated with increased melanoma risk, including ATM, MX2, and CASP8 (49,50). Furthermore, SNPs in the TERT and TRF1 genes, that play important roles in the regulation of telomerase activity and telomere length, are significantly associated with an increased melanoma risk (51). Interestingly, GWAS also found a novel TERT locus (TERT CLPTM1) that is positively associated with shorter telomere length and is inversely associated with the melanoma risk. Previously, it has been shown that there is a positive association between the number and size of nevi and telomere length (52,53). Few studies have addressed the role of telomerase in evasion of OIS and transformation of melanocytes, although recently Soo et al. reported that deregulation of telomerase function occurs late in the disease progression and not in early stages (54). Another susceptibility locus was identified at 1q21.3 that spans a region with ten genes, of which several are good candidate genes for melanoma susceptibility, including *ARNT* (Hif1β) and *SETDB1* (49,55). In fact, it was shown that SETDB1, which methylates histone H3 on lysine 9 (H3K9), significantly accelerates melanoma formation in zebrafish (56).

Additional advances in melanoma genetics have also been made with improved next generation sequencing technology. This allowed the comparison of the complete DNA sequence of cancer cells to their normal counterparts. Pleasance et al. catalogued the complete spectrum of somatic mutations and rearrangements in the metastatic cell line COLO-829 using a lymphoblastoid cell line from the same patient as a reference (57). This resulted in the identification of an astonishing total of 33,345 base substitutions of which 292 were somatic mutations (187 non-synonymous and 105 synonymous) in protein coding sequences. Since the ratio between non-synonymous and synonymous mutations did not significantly exceed random expectation, the majority of these mutations were catalogued as ‘passengers’. Interestingly, 70% of these mutations could be traced back to DNA damage induced by ultraviolet radiation, emphasizing its dominating role in melano magenesis. Previously identified driver mutations in *CDKN2A*, *BRAF*, and *PTEN* genes were confirmed in this study, while the oncogenes *GLI1*, *ETV5*, and tumor suppressor genes *DCC* and *TP63* were suggested as novel potential drivers. In addition, mutations were seen in the matrix metalloproteinase *MMP28* gene. Other novel but currently poorly characterized potential ‘driver’ genes included the transcription factor encoding SPDEF, the serine/threonine kinase 19 encoding STK19 and XIRP2, which encodes xin actin-binding repeat containing 2.

Collectively, GWAS and the next generation sequencing studies have identified several novel genes that might play a role in melanoma development and progression. However, extensive validation of these genes will be necessary to elucidate the mechanisms by which they contribute to melanocyte transformation. In order to perform such validations we must use and develop biologically adequate experimental models. Most of our current knowledge is based on *in vitro* studies, experimental grafting, and transgenic animal melanoma models. Difficulties in accessing relevant biological materials and recapitulating genetic complexity have hampered the direct analysis of disease progression in humans. This could possibly be overcome in the near future by the use of human induced pluripotent stem cells (hIPSCs). These pluripotent cells acquire most of the features of embryonic stem cells (ESCs) and can be differentiated into any cell type, including melanocytes (58).

Recently, Nissan et al. demonstrated that use of the cytokine bone morphogenic protein 4 allows generation of fully functional melanocytes from pluripotent stem cell lines of either induced or of embryonic origin (59). These melanocytes exhibit all the characteristic features of their adult counterparts, including the enzymatic machinery required for the production and functional delivery of melanin to keratinocytes. Furthermore, these cells integrate appropriately into an organotypic epidermis reconstructed *in vitro*. By applying such hIPSC technology, we will be able to obtain melanocytes from patients with genotypes of particular interest via a simple skin biopsy using easily accessible fibroblasts; hIPSC-derived melanocytes will retain the patients' complex genetic background, making it possible to study the impact of the genotype on disease development under a variety of experimentally controlled conditions, which would lead to a substantially better understanding of melanoma biology. Furthermore, using melanocytes in the correct genetic background would allow for testing targeted therapies within the context of proper host cells and thus provide new opportunities for target identification and validation.
